# Chemical Profiling of Polyphenolics in *Eucalyptus globulus* and Evaluation of Its Hepato–Renal Protective Potential Against Cyclophosphamide Induced Toxicity in Mice

**DOI:** 10.3390/antiox8090415

**Published:** 2019-09-19

**Authors:** Mosad A. Ghareeb, Mansour Sobeh, Walaa H. El-Maadawy, Hala Sh. Mohammed, Heba Khalil, Sanaa Botros, Michael Wink

**Affiliations:** 1 Medicinal Chemistry Department, Theodor Bilharz Research Institute, Kornaish El Nile, Warrak El-Hadar, Imbaba (P.O. 30), Giza 12411, Egypt; 2 Institute of Pharmacy and Molecular Biotechnology, Heidelberg University, 44883-2462 Heidelberg, Germany; sobeh@uni-heidelberg.de; 3 AgroBioSciences Research Division, Mohammed VI Polytechnic University, Lot 660–Hay MoulayRachid, 43150 Ben-Guerir, Morocco; 4 Pharmacology Department, Theodor Bilharz Research Institute, Kornaish El Nile, Warrak El-Hadar, Imbaba (P.O. 30), Giza 12411, Egypt; w.elmadawy@tbri.gov.eg (W.H.E.-M.); s.botros@tbri.gov.eg (S.B.); 5 Department of Pharmacognosy, Faculty of Pharmacy (Girls), Al-Azhar University, Cairo 11311, Egypt; halash1977@hotmail.com; 6 Pathology Department, Theodor Bilharz Research Institute, Kornaish El Nile, Warrak El-Hadar, Imbaba (P.O. 30), Giza 12411, Egypt; Seif200731@gmail.com

**Keywords:** *Eucalyptus globulus*, polyphenolics, cyclophosphamide, hepatotoxicity, nephrotoxicity, antioxidant, anti-inflammatory, Nrf2/HO-1 signaling

## Abstract

Cyclophosphamide (CP) is a potent anti-neoplastic and immunosuppressive agent; however, it causes multi-organ toxicity. We elucidated the protective activities of *Eucalyptus globulus* (EG) leaf extract against CP-induced hepato–renal toxicity. Mice were treated with EG for 15 days plus CP on day 12 and 13 of the experiment. Using HPLC-DAD-ESI-MS/MS, 26 secondary metabolites were identified in EG leaf extract. Out of them, 4 polyphenolic compounds were isolated: (1) 4-(*O*-β-d-xylopyranosyloxy)-3,5-di-hydroxy-benzoic acid, (2) 4-(*O*-α-l-rhamnopyranosyloxy)-3,5-di-hydroxy-benzoic acid, (3) gallic acid, and (4) methyl gallate. Effects of EG extract on biochemical parameters, gene expression, and immune-histopathological changes were assessed in comparison to mesna positive control. Results showed that EG improved CP-increased serum ALT, AST, creatinine, and blood urea nitrogen levels. The hepatic and renal tissue levels of MDA, nitric oxide, protein carbonyl, TNF-α, IL-6, and immunohistochemical expression of nuclear factor kappa-B (NF-kB) and caspase-3 were reduced. Also, hepatic and renal GSH contents, and nuclear factor E2-related factor 2 (NRf2)/ hemoxygenase-1 (HO-1) signaling levels were increased. Histopathological findings supported our findings where hepatic and renal architecture were almost restored. Results revealed the protective effects of EG against CP-induced hepato–renal toxicity. These effects may be related to EG antioxidant, anti-inflammatory, and anti-apoptotic properties coupled with activation of Nrf2/HO-1 signaling.

## 1. Introduction

Cyclophosphamide (CP) is prescribed in treating different types of neoplasms as well as an immunosuppressive drug in autoimmune diseases and organ transplantation [1,2]. Despite its chemotherapeutic efficacy and cost effectiveness, CP has a narrow therapeutic index and may be responsible for severe toxicity of vital organs [3,4]. Toxicity to liver and kidney are considered the two major ones, being the key organs responsible for CP metabolism and excretion, respectively [5,6]. CP is a prodrug that is metabolized within hepatocytes to generate two reactive metabolites, phosphoramide mustard and acrolein [7]. Phosphoramide mustard possesses antineoplastic activity, while acrolein is reported to be responsible for the CP-induced cytotoxic effects [8,9]. Acrolein initiates oxidative stress via the production of reactive oxygen species (ROS) causing the depletion of cellular defense mechanisms, induction of lipid peroxidation [10], nucleic acid damage and mutation [11]. The overproduction of ROS also induces several signaling molecules including nuclear factor kappa-B (NF-kB), which regulates the activation of different pro-inflammatory cytokines including interleukin (IL)-6, IL-1β and tumor necrosis factor (TNF)-α [12]. Moreover, CP is reported to down- regulate the stress sensor transcription factor nuclear factor erythroid 2-related factor 2 (Nrf2); one of the main defensive mechanisms against stress induced injuries [13,14].

CP is commonly administered with 2-mercaptoethane sulphonic acid (mesna); an adjuvant chemotherapeutic treatment regimen to counteract the prevalence of hemorrhagic cystitis and hematuria caused by the toxic metabolite acrolein [15]. However, the recurrence of hemorrhagic cystitis was reported with mesna [16] along with ineffectiveness in prevention of other reported CP-induced adverse drug reactions [15].

Because no adjuvant regimen is reported to offer protection to the healthy organs and tissues against CP toxicities without compromising its chemotherapeutic efficacy, enhancement of the antioxidant defense system to avert and/or reduce the adverse effects of CP and its reactive metabolites has been suggested [17]. Plant derived antioxidants preparations showed promising synergistic efficiency when co-administered with chemotherapeutic drugs mainly through their free radical scavenging and anti-inflammatory activities [18,19]. Natural antioxidants can enhance the current chemotherapeutic strategies not only by guarding against the adverse effects of chemotherapy but stimulating the host immune status as well [20].

*Eucalyptus globulus* Labill. (Myrtaceae) (EG) is an evergreen tree that is cultivated worldwide [21]. The leaf extract of EG exerts antimicrobial, antibacterial, anti-inflammatory, antioxidant, antihelmintic, and antiviral activities [22]. Moreover, previous studies reported that *Eucalyptus* extracts exhibited potent cytotoxic effects in various cell lines [23,24] and promising antitumor activity against Ehrlich ascites carcinoma in mice [25,26]. These properties could be related to the abundance of phenolic compounds in the extract such as caffeic acids, quinic, luteolin, dihydroxyphenylacetic, and hydrolysable tannins [27,28].

To the best of our knowledge, no previous study has investigated the hepatic and renal protective roles of EG against CP-intoxication. This study aims to identify and characterize the chemical profile of *E. globulus* (EG) and also investigate its protective role against CP-induced hepatic and renal toxicities in comparison to the adjuvant drug "mesna" by investigating the related mechanisms of action. 

## 2. Material and Methods

### 2.1. Drugs, Reagents and Instrumentations 

CP (Endoxan^®^) and mesna (Urometixan^®^) were purchased from Baxter Oncology GMBH, Halle, Germany. Dimethyl sulfoxide (DMSO) and phosphate buffered saline solution (PBS) were purchased from Sigma-Aldrich Chemical Co., MO, USA and Lonza Bio-products, Verviers, Belgium, respectively. All other solvents and reagents were of the highest grade commercially available. The ^1^H and ^13^C-NMR experiments were carried out using a BRUKER 400 MHz NMR spectrometer and samples were dissolved in deuterated DMSO-*d_6_*.

### 2.2. Preparation, Extraction and Fractionation of EG Leaf Extract as well as Chromatographic Isolation

Fresh leaves of EG were collected from Giza Governorate in June 2016. The identification and authentication of the collected plant was established by Dr. Tearse Labib, Botany Specialist, Department of Flora and Taxonomy, El-Orman Botanical Garden, Giza, Egypt.

Air dried leaves (1.5 kg) were extracted three times with methanol (4 L) at room temperature (25 ± 2 °C), the extract was concentrated via a rotatory evaporator to afford 220 g methanol extract. It was then defatted with 1.5 L petroleum ether (60–80 °C) to afford petroleum ether extract (25 g) and defatted methanol extract DME (175 g). DME (40 g) was subjected to polyamide (S6) column chromatography (CC) using polyamide column eluted with H_2_O/EtOH mixtures up to pure EtOH. By using PC, UV light and spray reagents, similar fractions were collected together to obtain three main fractions. Fraction (I) was purified via Sephadex LH-20/ EtOH & BIW (ethanol & butanol: isopropanol: water) to obtained compounds 1 and 2. Fraction II was subjected to successive CC on Sephadex LH-20/ EtOH & BIW to obtained compound 3. Fraction III was subjected to Sephadex LH-20 column and eluted via EtOH & BIW to yield compound 4.

### 2.3. HPLC-DAD-ESI-MS/MS Conditions

HPLC-DAD-ESI-MS/MS was employed to investigate the chemical constituents of the extract. The LC system was Thermo Finnigan (Thermo electron Corporation, OK, USA), coupled with an LCQ Duo ion trap mass spectrometer with an ESI source (ThermoQuest). A Silica gel C18 reversed-phase column (Zorbax Eclipse XDB-C18, Rapid resolution, 4.6 × 150 mm, 3.5 µm (Agilent, CA, USA) was used for the separation process. Water with a gradient increase from 5% to 50% of acetonitrile (ACN) (with 1% formic acid each in the positive mode) was applied in 60 min, with a flow rate 1 mL/min, and then increased to 90% ACN in the next 30 min. The samples were injected automatically using auto sampler surveyor ThermoQuest. The instrument was controlled by Xcalibur software (Thermo Fisher Scientific Inc., OK, USA). The MS operating conditions were applied in the negative ion mode, as previously described by us [29]. The ions were detected in a full scan mode and mass range of 50–2000 *m/z*.

### 2.4. Animals 

Adult male Swiss albino mice weighing between 25 ± 5 g (purchased from the Schistosome Biology Supply Center, Theodor Bilharz Research Institute, Giza, Egypt) were used. Mice were kept in the animal house facility of the institute in standard polypropylene cages at 25 ± 2 °C temperature with 50–60% relative humidity, 12 h light–dark cycles with free access to ad libitum food and water. The study protocol was approved by the Research Ethics Committee of Theodor Bilharz Research Institute (PT: 19/2/467). All procedures were conducted in accordance with the guidelines of the National Institutes of Health (NIH, 1996) and its amendments for the care and use of laboratory animals.

### 2.5. Experimental Design

Mice were randomly allocated into five groups each of six animals. Group 1 (control): Received vehicle for EG (0.5% DMSO in PBS solution) for 15 consecutive days; Group 2 (CP): Received CP in PBS (200 mg/kg, [30] on day 12 and 13 of the experiment, Groups 3 (CP + mesna): Received the positive control mesna (40 mg/kg, [31] 1 h before and 4 h after each CP application, Groups 4 and 5 (CP + EG): Received EG in doses of 50 and 100 mg/kg [25,26], respectively once daily for 15 days along with CP (on day 12 and 13). All treatments were administered intraperitoneally (i.p.).

After the end of treatments mice were killed under anesthesia. Blood, kidney, and liver samples were collected. Sera were separated by centrifugation and stored at −80 °C until analysis. The livers and kidneys were washed in cold PBS; sections of livers and kidneys were fixed in 10% neutral buffered formalin for histopathological and immunohistochemical examinations. The remaining tissue samples were homogenized (10% *w/v*) in cold PBS for biochemical analyses.

### 2.6. Determination of Liver and Kidney Toxicity Indices

Serum levels of alanine aminotransferase (ALT), aspartate aminotransferase (AST), creatinine, and blood urea nitrogen (BUN) were spectrophotometrically determined using commercially available kits (Biodiagnostics, Cairo, Egypt).

### 2.7. Determination of Oxidative/Nitrosative Stress Markers and Protein Carbonyl in Liver and Kidney Tissues

The supernatants of homogenized liver and kidney samples were used for determination of reduced glutathione (GSH) and lipid peroxidation (MDA) levels according to Ellman, 1957 [32] and Ohkawa et al., 1979 [33], respectively. The protein carbonyl (PC) (OxiSelect Cell Biolabs, CA, USA) and nitric oxide (NO) levels (Biodiagnostics, Cairo, Egypt) were measured using commercially available kits and the amount of total protein was determined by BCA protein assay kit (Thermo Fischer Scientific, IL, USA).

### 2.8. Determination of Nrf2/HO-1 Pathway Activation in Liver and Kidney Tissues

Gene expression levels of Nrf2 were determined using quantitative reverse transcriptase real time polymerase chain reaction (qRT-PCR). Briefly, total RNA was isolated from hepatic and kidney samples using TRIzol (Invitrogen, CA, USA), and quantified using a nanodrop. RNA samples were used for DNA synthesis using cDNA Synthesis Kit. Amplification of the cDNA was carried out by SYBR Green master mix (Applied biosystems, CA, USA). All values were normalized to the housekeeping β-actin gene. Relative expression of studied genes was calculated using the comparative threshold cycle method (2^−ΔΔCt^ method).

The sequences of PCR primer pairs used were: 

Nrf2 F: TTGTAGATGACCATGAGTCGCR: TGTCCTGCTGTATGCTGCTTΒ-actin F: AGGAGTACGATGAGTCCGGCR: CGCAGCTCAGTAACAGTCCG

Next, the hepatic and renal levels of heme oxygenase-1 (HO-1) were measured using the commercial ELISA kit according to the manufacturer’s instructions (OxiSelect Cell Biolabs, CA, USA). 

### 2.9. Determination of Pro-inflammatory Markers and Caspase-3 in Liver and Kidney Tissues

The liver and kidney pro-inflammatory markers, TNF-α and IL-6, were measured using the commercially available ELISA kits according to the manufacturer’s instructions (OxiSelect Cell Biolabs, CA, USA) and the amount of total protein was determined by a BCA protein assay kit.

Moreover, sections on charged slides (Superfrost charged slides, Thermo Scientific, Braunschweig, Germany) from liver and kidney tissues were immunohistochemically stained with anti-NF-κB and anti-caspase-3 (Santa Cruz Biotechnology, CA, USA, respectively) using an Ultra Benchmark machine (Roche, Tucson, USA) and an Optiview Detection kit with haematoxlin as the counterstain. The percent of positively stained brown nuclei and cytoplasm for NF-κB and caspase-3, respectively, were examined in five microscopic fields (at x400 under Zeiss light microscopy, Jena, Germany).

### 2.10. Histopathological Examination

Liver and kidney tissues embedded in paraffin blocks were sectioned at 4 µm thickness. Sections were stained with hematoxylin/eosin (H&E) and blindly examined for the extent of liver and kidney damage under bright field microscope (Olympus BX53F).

### 2.11. Statistical Analysis

Data are expressed as mean ± SEM. Statistical analysis was performed using one-way analysis of variance (ANOVA) followed by Tukey–Kramer as post hoc test for multiple comparisons (GraphPad Software, San Diego, CA, USA, version 5.03). *p* < 0.05 was considered statistically significant.

## 3. Results

### 3.1. HPLC-DAD-ESI-MS-MS Annotation and Chromatographic Isolation of Polyphenolic Compounds

**Figure 1:**
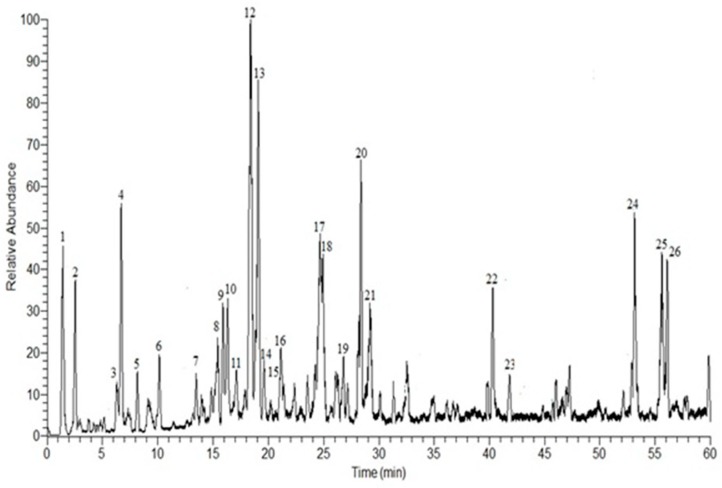
Negative HPLC-DAD-ESI-MS/MS profile of phenolic compounds from the methanol extract of *E. globulus* leaves. Peak numbers agree with those in Table 1.

**Figure 2:**
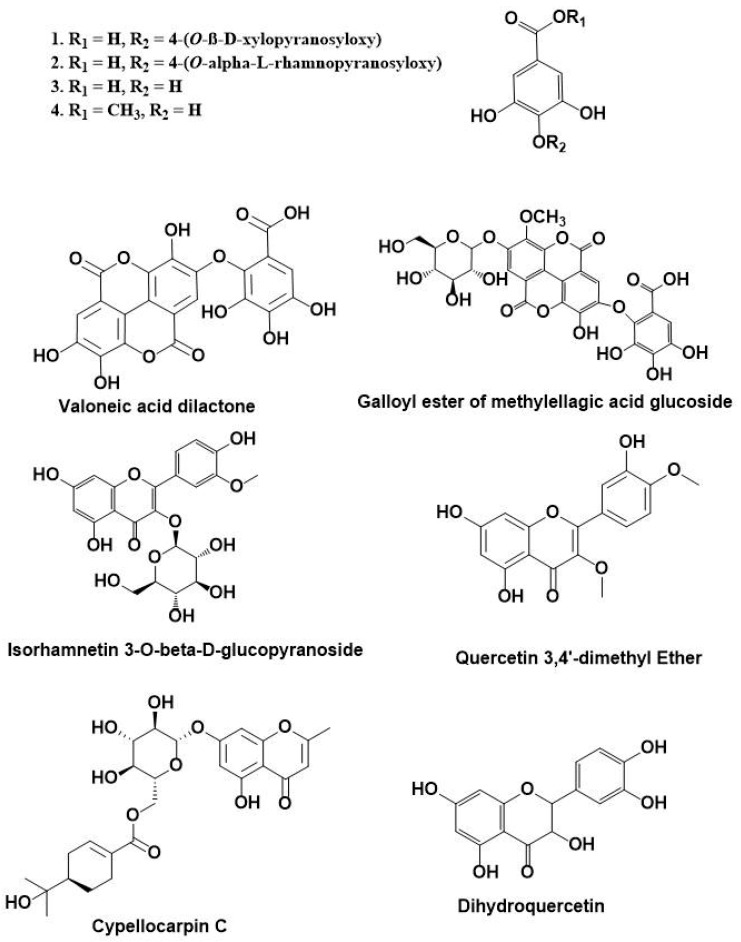
Chemical structures of phenolic compounds isolated from *Eucalyptus globulus* as well as some major annotated compounds by HPLC-DAD-ESI-MS/MS.

In the current study, HPLC-DAD-ESI-MS/MS was used to identify the polyphenolic secondary metabolites in the methanol extract of *E. globulus* leaves. Based on the retention time and fragmentation pattern in the negative ion mode, 26 compounds were tentatively identified in the extract and were categorized as phenolic acids, flavonoids, and hydrolyzable tannins. The major identified compounds were gallic acid pentoside (1), gallic acid rhamnoside (2), gallic acid (3), methyl gallate (4), isorhamnetin 3-*O*-β-d-glucuronoside (12), galloyl cypellocarpin B (13), galloyl ester of a methylellagic acid glucoside (17), quercetin-3,4’-dimethyl ether (18), cypellocarpin C (20), dihydroquercetin (22), valoneoyl-digalloyl-glucopyranose (24), eicosanoic acid (25), and valoneic acid dilactone (26) (Supplementary data S1). The results are documented in Table 1 and Figure 1. Out of the annotated compounds, 4 compounds were isolated and characterized based on their NMR data as (1) 4-(*O*-β-d-xylopyranosyloxy)-3,5-di-hydroxy-benzoic acid, (2) 4-(*O*-α-l-rhamnopyranosyloxy)-3,5-di-hydroxy-benzoic acid, (3) 3,4,5-trihydroxybenzoic acid (gallic acid), and (4) methyl 3,4,5-trihydroxybenzoate (methyl gallate) (Figure 2) (Supplementary data S2).

### 3.2. EG Pretreatment Alleviated CP Induced Liver and Kidney Damage

CP caused a substantial increase in serum levels of ALT, AST, creatinine, and BUN (3, 3.6, 1.9, and 3.4 fold, respectively) when compared to normal controls. Mesna modestly reduced their levels when compared to CP treated animals. Pretreatment with EG at doses of 50 and 100 mg /kg resulted in a significant dose dependent reduction in these parameters when compared to corresponding CP or mesna treated mice. EG when given in a dose of 100 mg/kg normalized the levels of ALT, AST, and creatinine (Table 2).

**Figure 3:**
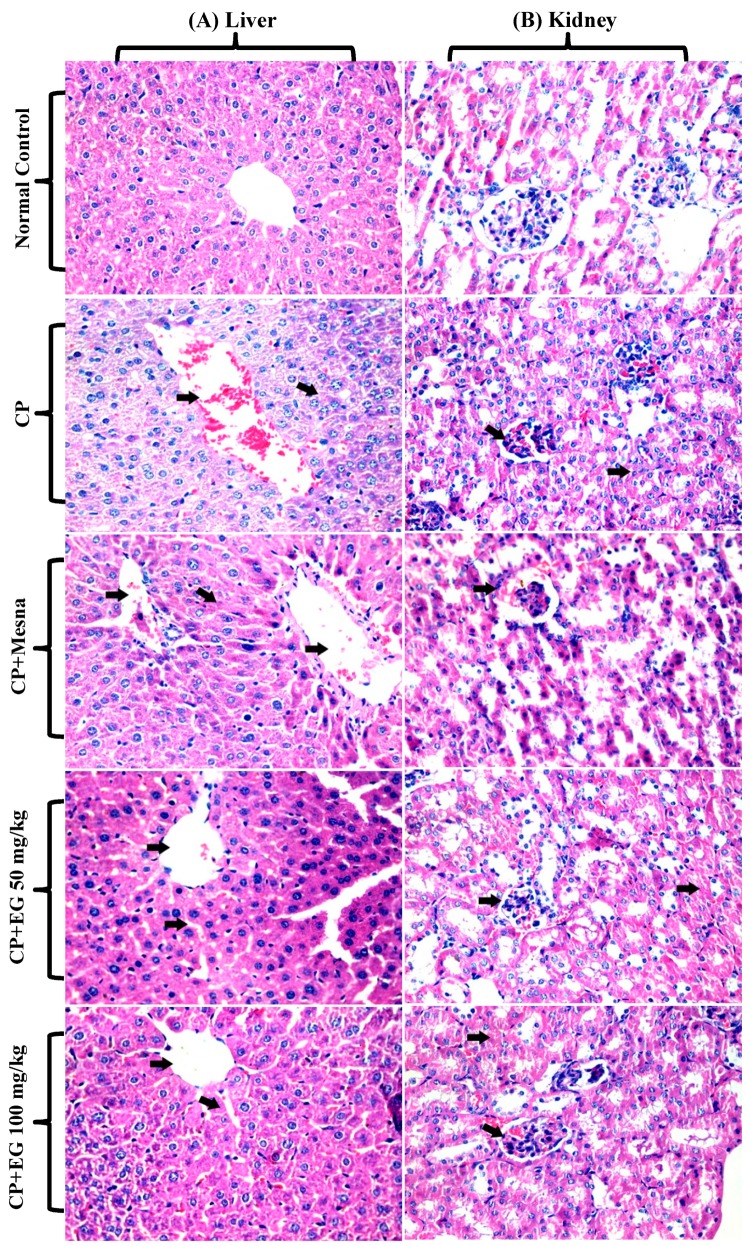
Photomicrographs showing the protective effects of EG pretreatment on CP–induced damage in hepatic (**A**) and renal (**B**) tissue sections stained with H&E (×400).

Hepatic tissues of CP treated mice revealed hydropic degeneration of hepatocytes, hyperemia, congestion, dilatation of sinusoids. Minor to moderate improvement in hepatic damage was recorded in mesna and EG (50 mg/kg) treated mice, respectively, whereas normal hepatic architecture was recorded upon pretreatment with 100 mg/kg EG (Figure 3A). Renal tissues of CP treated mice showed degenerated renal tubules with hyaline casts, atrophy in the glomeruli with mesangeal proliferation, tubular degeneration and dilatation in bowman capsules. Pretreatment with EG in doses of 50 and 100 mg/kg revealed a dose dependent improvement in the histological changes of the glomeruli and renal tubules (Figure 3B).

### 3.3. EG Pretreatment Mitigated CP-induced Oxidative/Nitosative Stress and Protein Carbonylation in Liver and Kidney Tissues

**Figure 4:**
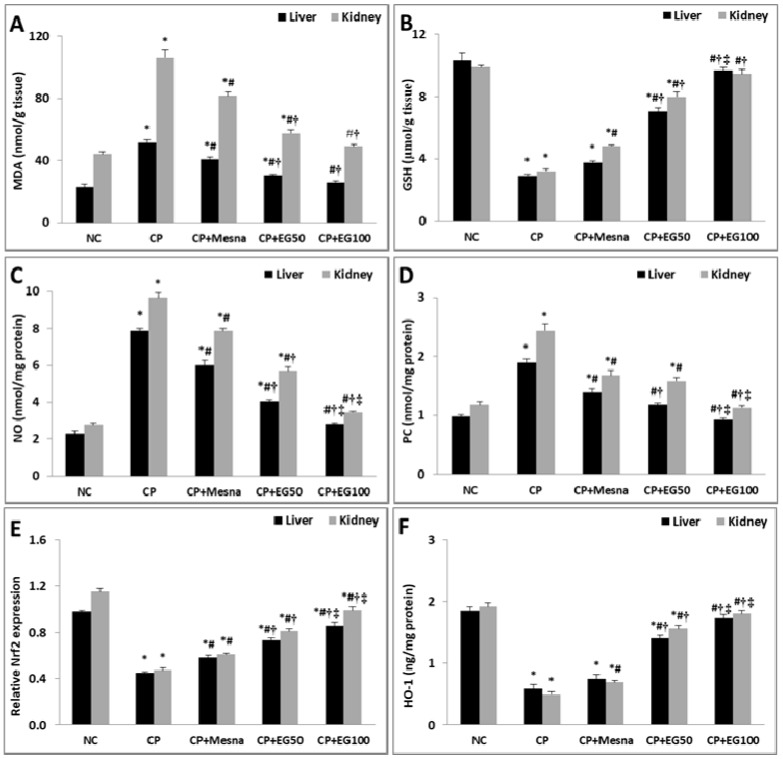
Effect of EG pretreatment on hepatic and renal levels of MDA (**A**), GSH (**B**), NO (**C**) and PC (**D**), on Nrf2 gene expression (**E**) and HO-1 levels (**F**) in CP-treated mice.

CP caused a considerable increase in the liver and kidney MDA levels (2.3- and 2.4-fold, respectively) with depletion in their GSH contents (71.7 and 67.6%, respectively) as compared to normal controls. Mesna revealed insignificant changes in hepatic GSH content, trivial decrement in the increased MDA levels, but resulted in significant improvement in renal GSH contents (50.6%) in comparison to CP treated mice. Compared to CP or mesna treated mice, pretreatment with 50 mg/kg EG resulted in significant drop in the hepatic and renal MDA levels with marked increase in their GSH contents. Pretreatment with 100 mg/kg EG restored their normal levels in both the hepatic and renal tissues (Figure 4A,B).

Compared to normal mice, CP induced a marked increase in NO and PC levels of both hepatic (3.5- and 1.9-fold, respectively) and renal (3.5- and 2-fold, respectively) tissues. Mesna showed a significant decrease in their levels in hepatic (23.8 and 26.80%, respectively) and renal (18.7 and 31.2%, respectively) tissues when compared to CP treated animals. Pretreatment with either 50 or 100 mg/kg EG notably alleviated the increased levels of PC and NO in the liver and kidney in a dose dependent way when compared to CP and mesna treated mice. Pretreatment with 100 mg/kg EG neutralized the increased PC and NO levels in the liver and kidney of CP treated mice (Figure 4C,D).

Data are represented as mean ± SEM (*n* = 6). ^*^ *p* < 0.05 vs. normal control, ^#^ *p* < 0.05 vs. CP, ^†^ *p* < 0.05 vs. CP + mesna, ^‡^ *p* < 0.05 *vs* CP + EG (50 mg/kg). Statistical analysis was done using one-way ANOVA followed by Tukey’s multiple comparisons test. MDA: malondialdehyde, GSH: reduced glutathione, NO: nitric oxide, PC: Protein carbonyl, Nrf2: nuclear factor erythroid 2-related factor 2, HO-1: heme oxygenase-1, NC: normal control, CP: cyclophosphamide, EG: *E. globulus*.

### 3.4. EG Pretreatment Activated Nrf2/HO-1/Antioxidant Signaling in The Livers and Kidneys of CP Treated Mice

CP treated mice showed a substantial drop in hepatic and renal gene expression of Nrf2 (54 and 59.1%, respectively) in comparison to normal controls. Mesna demonstrated a moderate decline in hepatic (28.9%) and renal (29.8%) expression of Nrf2 as compared to CP treated mice. Mice treated with either 50 or 100 mg/kg EG exhibited a considerable rise in the hepatic (62.2 and 91.1%, respectively) and renal (72.3 and 110.6%, respectively) Nrf2 mRNA expression when compared with CP treated animals. Moreover, the enhancement in the hepatic (25.86 and 48.28%, respectively) and renal (32.79 and 62.30%, respectively) Nrf2 expression was more prominent when compared to mesna treated groups (Figure 4E).

The cytoprotective isoenzyme HO-1 levels were significantly reduced in hepatic (68.7%) and renal (74.5%) tissues of CP treated mice when compared to normal controls. Mesna did not reveal any significant changes in hepatic HO-1 levels whereas a significant rise of renal HO-1 levels (40.8%) was recorded in comparison with CP treated groups. Mice pretreated with EG in a dose of 50 and 100 mg/kg showed a dose dependent increase in hepatic HO-1 levels when compared to either CP (2.4- and 3-fold) or mesna (1.9- and 2.3-fold) treated groups, respectively. Similarly, a dose dependent elevation in renal HO-1 levels was detected in pretreated groups with EG (50 and 100 mg/kg) when compared to either CP (3.2- and 3.7-fold) or mesna (2.3- and 2.6-fold) treated groups. Moreover, pretreatment with 100 mg/kg EG normalized the reduced HO-1 levels in livers and kidneys of CP-treated mice (Figure 4F). 

### 3.5. EG Pretreatment Down-regulated CP-induced Inflammation in Liver and Kidney Tissues

**Figure 5:**
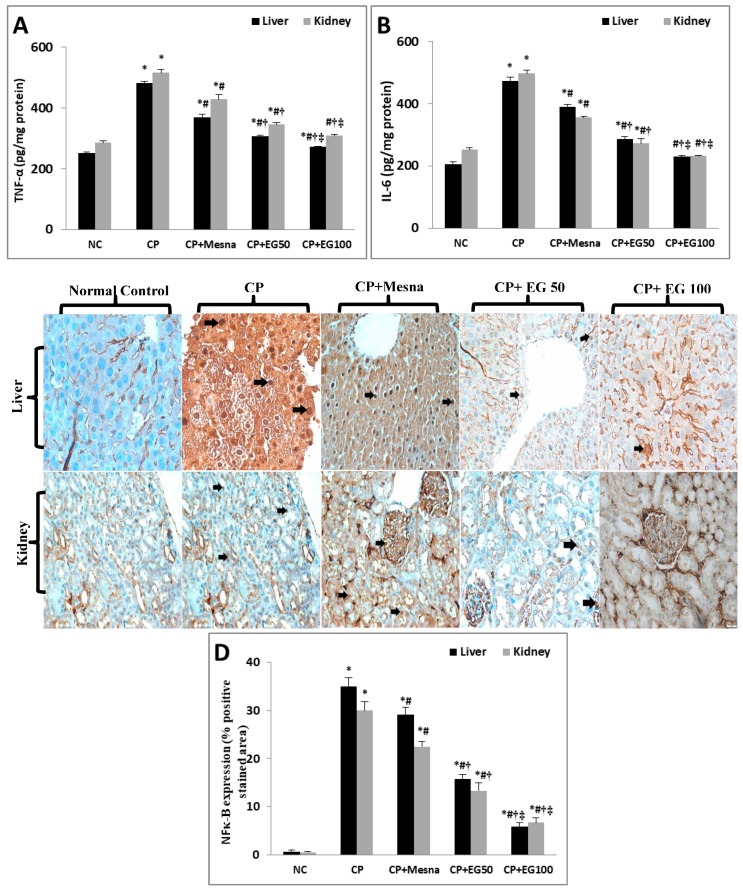
Effect of EG pretreatment on liver and kidney levels of pro-inflammatory markers (TNF-α (**A**), IL-6 (**B**)), IHC expression of NF-κB (×400, black arrows represent localization of positively stained brown nuclei with faint ignored background staining) (**C**) and semi-quantitative estimation of NF-κB positively stained nuclei in hepatic and renal tissues of CP-treated mice.

The levels of the pro-inflammatory cytokines, TNF-α and IL-6, were increased in the livers (92.1% and 129.9%) and kidneys (80.8% and 97.8%) of CP treated mice when compared to normal controls. Mesna caused a partial reduction in both hepatic and renal levels of TNF-α (23.6% and 17%) and IL-6 (17.7% and 28.5%) when compared to CP treated groups. Pretreatment with EG resulted in a dose dependent reduction in their hepatic and renal levels in comparison to either CP or mesna treated groups. Additionally, EG pretreatment in a dose of 100 mg/kg neutralized the elevated hepatic and renal levels of both cytokines (Figure 5A,B).

IHC expression of NF-κB revealed minimal basal levels in normal hepatic and renal tissues. However, a prominent up-regulation in its expression was detected in CP-treated liver (mainly in hepatocytes around the central vein) and kidney (identified in the renal tubules and glomeruli) tissues by 52.2- and 60-fold, respectively, as compared to normal controls. Mesna showed a significant reduction by 16.7% and 25%, respectively, when compared to the CP-treated group. EG pretreatment in doses of 50 and 100 mg/kg exhibited a dose dependent decline in hepatic expression of NF-κB in comparison to CP (54.8% and 83.4%, respectively) and mesna (45.7% and 80%, respectively) treated groups. A similar dose dependent down-regulation was observed in NF-κB renal expression in comparison to CP (55.6% and 77.8%, respectively) and mesna (40.7% and 70.4%, respectively) treated groups (Figure 5C,D).

Data are represented as mean ± SEM (*n* = 6). ^*^ *p* < 0.05 vs. normal control, ^#^ *p* < 0.05 vs. CP, ^†^ *p* < 0.05 vs. CP + mesna, ^‡^ *p* < 0.05 vs. CP + EG (50 mg/kg). Statistical analysis was done using one-way ANOVA followed by Tukey’s multiple comparisons test. TNF-α: tumor necrosis-α, IL-6: interleukin-6, NF-κB: nuclear factor-kappa B, NC: normal control, CP: cyclophosphamide, EG: *E. globulus*.

### 3.6. EG Blocked CP-induced Apoptosis in the Liver and Kidney of Mice

**Figure 6:**
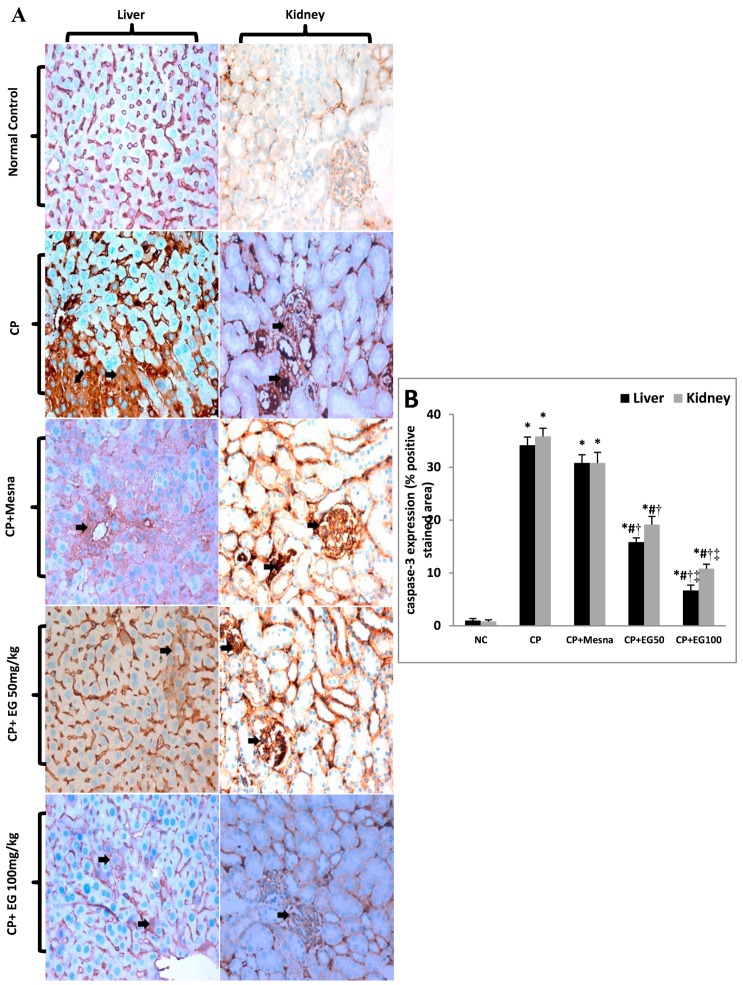
Effect of EG pretreatment on IHC expression of caspase-3 (×400, black arrows represent localization of positively stained brown cytoplasm with ignored cross reaction in sinusoids as appeared in normal control section) (**A**) and semi-quantitative estimation of caspase-3 positively stained cells in hepatic and renal tissues of CP-treated mice.

IHC expression of caspase-3 was markedly up-regulated in CP treated hepatic (34.2-fold) and renal tissues (43.2-fold) when compared to untreated tissues. Caspase-3 positively stained cells were observed mainly in hepatocytes around the central vein and in the renal tubules and glomeruli. Administration of mesna did not show significant changes in hepatic and renal caspase-3 expression when compared to CP-treated groups. EG pretreatment in doses of 50 and 100 mg/kg showed dose dependent reduction in hepatic expression of caspase-3 in comparison to CP (53.7% and 80.5%, respectively) and mesna (48.7% and 78.4%, respectively) treated groups. Also, a dose dependent down-regulation in caspase-3 renal expression was observed in comparison to CP (46.5% and 69.8%, respectively) and mesna (37.8% and 64.9%, respectively) treated groups (Figure 6A,B).

Data are represented as mean ± SEM (*n* = 6). ^*^ *p* < 0.05 vs. normal control, ^#^ *p* < 0.05 vs. CP, ^†^ *p* < 0.05 vs. CP + mesna, ^‡^ *p* < 0.05 vs. CP + EG (50 mg/kg). Statistical analysis was done using one-way ANOVA followed by Tukey’s multiple comparisons test. NC: normal control, CP: cyclophosphamide, EG: *E. globulus.*

## 4. Discussion

In this study, the hepato–renal protective activities of EG were examined in CP treated mice. Results were compared to the uroprotective thiol “mesna”, which is routinely prescribed as an adjuvant treatment regimen to reduce the risk of hemorrhagic cystitis [15].

In this study, CP treated mice revealed a prominent increase in serum levels of hepatoxicity biomarkers (ALT and AST) by approximately 3-fold, as previously described [17,49]. Hepatotoxicity is reported to be one of the major side effects of CP induced hepatic damage with increased permeability of cell membrane and leakage of liver enzymes, specifically ALT and AST [50]. EG did not only normalize the elevated levels of ALT and AST but also restored the normal hepatic architecture. In CP treated mice, impairment in kidney functions was expressed as increased leakage of creatinine and urea into the systemic circulation, glomerular degenerative changes, and atrophy in kidney tissues. The recorded steep reduction in the levels of creatinine and BUN accompanied with amelioration of the kidney degenerative changes in EG pretreated groups may point to a possible nephro-protective activity.

In the current study, the oxidative/antioxidative mechanisms were examined, since oxidative stress is well known to play a pivotal role in the pathogenesis of CP induced toxicity, which mainly results from its toxic metabolite acrolein [51]. Acrolein induces the generation of ROS leading to enhanced lipid peroxidation and reduction in the antioxidant defense system in liver and kidney tissues [14,52]. In agreement with previous studies [17,49], our data showed an exacerbation in ROS production as denoted by the significant increase in the final product of lipid peroxidation, MDA, in liver and kidney of CP treated mice. Moreover, a substantial depletion in hepatic and renal GSH contents (by 72% and 68%, respectively) was observed. This could be attributed to the direct conjugation of CP and its metabolites (acrolein and phosphoramide mustard) to the free or protein-bound –SH groups of GSH [53], denoting declined cellular defenses [54]. Pretreatment with EG restored the deficient thiol store in hepatic and renal cells by promoting the de novo synthesis of GSH. Previous studies documented the free radical scavenging activities of EG in alloxan-induced oxidative stress [55] and acetaminophen-induced kidney damages in rats [56]. In this study HPLC-DAD-ESI-MS/MS analysis of EG leaf extract revealed the presence of polyphenols with flavonoids (luteolin, kampferol and iridin) and tannins (gallotannins and ellagitannins) as major constituents. Flavonoids [29,57] and tannins [58] are reported to exhibit powerful antioxidant and free radical scavenging effects. 

NO is known to play an essential role in regulating cellular stress [59]. NO directly result from the up-regulated expression of inducible NO synthase (iNOS) indicating impaired cellular viability [60]. NO is apparently involved in CP induced toxicity [59,61], this is in coherence with our findings where NO was significantly increased in both the hepatic and renal tissues of CP treated mice by almost 3-fold. Moreover, the detected increase in NO was accompanied with elevated liver and kidney PC contents in CP treated mice by almost 2-fold. PC content is a marker expressing the extent of protein damage [62,63], and its enhanced levels is indicative of DNA damage and mutation resulting from excessive production of ROS in liver [64] and kidney [65] tissues. EG pretreatment led to normalization of NO levels indicating hepato- and nephro-protection against CP induced oxidative stress. In addition, the reduced hepatic and renal PC contents may indicate significant recovery of DNA damage suggesting possible assistant role for EG in tissue regeneration.

In addition, we investigated the impact of EG pretreatment on the up-regulation of the Nrf2/HO-1/antioxidant signaling pathway, which is considered one of the key defense mechanisms against stress-associated injuries [66]. Under stress conditions, various stimuli as ROS and pro-inflammatory cytokines [67] adversely result in the down-regulation of Nrf2 expression in liver and kidney cells, thereby moderating the transcription of the cytoprotective isoenzyme heme oxygenase-1 (HO-1) [14,19]. In the current work, CP resulted in a significant down-regulation of the hepatic and renal Nrf2 gene expressions along with marked reduction in their HO-1 levels, which could be linked to the excessive production of ROS, as previously reported [68,69]. Notably, pretreatment with EG demonstrated an activation of Nrf2 gene expression complemented with subsequent elevation in HO-levels in both hepatic and renal tissues implying that the recorded EG antioxidant effects can be partly attributed to the up-regulation of the Nrf2/HO-1 antioxidant signaling pathway. 

Additionally, the oxidative/nitrosative stress generated by CP is known to be associated with the subsequent activation of inflammatory cascades [19,30]. NF-κB is a redox-sensitive transcription factor that regulates the expression and activation of pro-inflammatory cytokines and other mediators of inflammation including IL-6, TNF-α, and iNOS [70,71,72]. In the current study, CP-treatment elicited an increase in NF-κB IHC expression with marked elevation in levels of TNF-α and IL-6 in hepatic and renal tissues [14,60]. The increased NF-kB expression is known to trigger the expression of iNOS, which in turn causes the overproduction of NO [73], as reported herein. The recorded decrease in the hepatic and renal levels of the pro-inflammatory cytokines (TNF-α and IL-6), NO along with subsequent down-regulation in NF-κB expression in EG pretreated mice could be related to EG compensatory mechanism against the inflammatory milieu generated in CP treated mice. The anti-inflammatory activities of EG was previously demonstrated in inflammatory-mediated disorders [74].

CP induced oxidative/nitrosative stress and inflammatory responses are also known to trigger apoptotic cell death via both mitochondria-dependent and mitochondria-independent apoptotic pathways [75], leading to activation of the executioner apoptotic marker caspase-3 [76]. The IHC expression of caspase-3 was up-regulated in both hepatic and renal tissues of CP treated mice. Pretreatment with EG extract counteracted the CP-induced apoptotic changes as evidenced by the marked regression in caspase-3 positively stained cells. This observed anti-apoptotic effect of EG could be directly linked to its antioxidant and anti-inflammatory properties.

In this study, mesna demonstrated minor to moderate hepatoprotective and nephroprotective activities. EG showed better results in terms of dose dependent improvement in the assessed biochemical and imunohistopathological parameters upon the use of EG, especially when the dose of 100 mg/kg was applied. These findings are in agreement with US FDA report (2009) [15] stating the ineffectiveness of mesna in preventing or ameliorating other reported multi-organ toxicity elicited by CP. Our findings may be explained in the view of the pharmacokinetic profile of mesna, which is known to be distributed in the body in its biologically inactive disulfite form. It passes through the hepatic vasculature in an unchanged form [77] and undergoes reduction in the renal epithelial cells to the pharmacologically active thiol form, mesna, which is then excreted in urine and combines with acrolein to form the stable uro-nontoxic compounds via its sulfhydryl group [78]. 

## 5. Conclusions

HPLC-MS/MS profiling of the tannins-rich leaf extract of *E. globulus* resulted in characterization of 26 secondary metabolites including tannins, phenolic acids, and flavonoids. Moreover, our results revealed that alleviation of CP-induced hepato/renal-toxicities can be related to the augmentation of antioxidant defenses at least partially through induction of Nrf2/HO-1 signaling with attenuation of excessive inflammatory responses as well as apoptosis in the hepatic and renal tissues. EG may represent an effective and economic plant product that can protect against the risks of toxic CP activities. However, further investigations are required to examine the potential synergistic activity of EG on the chemotherapeutic efficacy of CP. Also, investigations are required in a clinical context to confirm its hepato- and nephro-protective activities. 

## Figures and Tables

**Table 1:** Phenolic compounds tentatively identified in the methanol extract of *E. globulus* (EG) leaves by HPLC-DAD-ESI-MS/MS.

No.	*R_t_*	[M-H]^−^	Major Product Ions (*m/z*)	Tentative Identified Compounds *
1	1.41	301	**169, 125** ^c^	Gallic acid pentoside ^a,b^
2	2.54	315	**169, 125**	Gallic acid rhamnoside ^a,b^
3	6.30	169	**125**	Gallic acid ^a,b^
4	6.69	183	**183**, 169, 125	Methyl gallate ^a,b^
5	8.16	353	191, 161	Chlorogenic acid
6	10.46	267	251, 223, **221**, 205, 203, 193, 97, 85	Unidentified
7	13.55	537	313, **271**, 211, 169	Mallophenol B
8	15.89	521	491, 359, 179	Rosmarinic acid hexoside
9	16.29	483	331, **271**, 211, 169	Digalloylglucose
10	17.16	421	331, 313, **169**, 151, 125	Benzyl-galloylglucose
11	17.50	481	463, 301, **271**, 151	Hexahydroxydiphenoyl-glucose
12	18.68	491	473, 431, **315**, 301, 179	Isorhamnetin 3-*O*-β-d-glucuronoside
13	19.64	689	**537**, 519, 211, 193	Galloyl cypellocarpin B
14	19.82	939	**769**, 635, 617, 599, 465	Pentagalloylglucose
15	20.12	635	**483**, 465, 423, 331, 169	Trigalloylglucose
16	21.54	625	473, 463, 437, 301, 257	HHDP-diglucoside
17	24.91	629	477, 315, **301**	Galloyl ester of a methylellagic acid glucoside
18	25.31	329	329, **314**, 301, 300, 299, 285, 243	Quercetin-3,4’-dimethyl ether
19	27.17	1085	765, 633, 473	Eucalbanin A or cornusiin B
20	28.52	519	353, 335, **233**	Cypellocarpin C
21	29.61	1415	1113, 933, 783, 633	Di (HHDP-galloylglucose)-pentose
22	40.34	303	301, 285, **259**, 179, 125	Dihydroquercetin (Taxifolin)
23	41.82	617	**465**, 343, 303, 169	Trigalloyllevoglucosan
24	53.13	953	635, 301, 169	Valoneoyl-digalloyl-glucopyranose
25	55.60	311	**296**, 293, 195	Eicosanoic acid
26	56.04	469	425, **423**, 301, 169	Valoneic acid dilactone

* HPLC-DAD-ESI-MS-MS annotation of the polyphenolic compounds based on their retention time, fragmentation pattern, and via comparison of MS spectra with the reported data [34,35,36,37,38,39,40,41,42,43,44]. ^a^ Compounds isolated and identified via HPLC-DAD-ESI-MS/MS during this study. ^b^ The structural elucidation of the isolated phenolic compounds (1–4) was based on spectral and chemical analyses as well as literature [45,46,47,48]. ^c^ Bold items referred to the main aglycones fragments.

**Table 2:** Protective effects of EG pretreatment on the serum markers against cyclophosphamide (CP)-induced hepato–renal toxicities in mice.

Animal Groups	Liver Functions		Kidney Functions	
	ALT	AST	Creatinine	BUN
Normal Control	23.65 ± 0.80	42.38 ± 1.27	0.26 ± 0.02	11.97 ± 0.47
CP	70.17 ± 1.65 *	152.39 ± 1.34 *	0.49 ± 0.02 *	40.40 ± 1.71 *
CP + mesna	60.11 ± 1.60 *^#^	126. 26 ± 1.33 *^#^	0.39 ± 0.01 *^#^	31.71 ± 0.91 *^#^
CP + EG (50 mg/kg)	33.68 ± 0.90 *^#†^	61.11 ± 1.23 *^#†^	0.34 ± 0.01 *^†^	25.68 ± 0.65 *^#†^
CP + EG (100 mg/kg)	24.78 ± 0.65 ^#†‡^	46. 10 ± 0.96 ^#†‡^	0.29 ± 0.01 ^#†‡^	16.29 ± 0.52 *^#†‡^

Data are represented as mean ± SEM (*n* = 6). ^*^ *p* < 0.05 vs. normal control, ^#^ *p* < 0.05 vs. CP, ^†^ *p* < 0.05 vs. CP+mesna, ^‡^ *p* < 0.05 vs. CP+EG (50 mg/kg). Statistical analysis was done using one-way ANOVA followed by Tukey’s multiple comparisons test. ALT: alanine aminotransferase, AST: aspartate aminotransferase, BUN: blood urea nitrogen, CP: cyclophosphamide, EG: *E. globulus*.

## Supplementary Materials

The following are available online at https://www.mdpi.com/2076-3921/8/9/415/s1. Annotation of the polyphenolic compounds using HPLC-DAD-ESI-MS/MS (S1) and structural elucidation of the isolated phenolic compounds (S2).

## Abbreviations

HPLC-DAD-ESI-MS/MS	High performance *liquid chromatography* coupled with diode array detection (*DAD*) and electrospray *mass spectrometry* (MS)
EG	Eucalyptus globulus
CP	Cyclophosphamide
NF-κB	Nuclear factor kappa-B
PC	Protein carbonylation
Nrf2	Nuclear factor E2-related factor 2
HO-1	Hemoxygenase-1
(IL)-6	Interleukin
(TNF)-α	Tumor necrosis factor
ROS	Reactive oxygen species
Mesna	(2-mercaptoethane sulphonic acid)
DMSO	Dimethyl sulfoxide
PBS	Phosphate buffered saline
DME	Defatted methanol extract
PC	Paper chromatography
UV	Ultra-violet
CC	Column chromatography
BIW	Butanol: isopropanol: water
i.p.	Intraperitoneally
*ALT*	Alanine transaminase
*AST*	Aspartate transaminase
BUN	Blood urea nitrogen
qRT-PCR	Quantitative reverse transcriptase real time polymerase chain reaction
H&E	Hematoxylin/eosin
MDA	Malondialdehyde
iNOS	Inducible nitric oxide synthase
US FDA	United States Food and Drug Administration
NO	Nitric oxide
