# Correction: Ghareeb et al. Chemical Profiling of Polyphenolics in *Eucalyptus globulus* and Evaluation of Its Hepato–Renal Protective Potential Against Cyclophosphamide Induced Toxicity in Mice. *Antioxidants* 2019, *8*, 415

**DOI:** 10.3390/antiox14091098

**Published:** 2025-09-09

**Authors:** Mosad A. Ghareeb, Mansour Sobeh, Walaa H. El-Maadawy, Hala Sh. Mohammed, Heba Khalil, Sanaa Botros, Michael Wink

**Affiliations:** 1 Medicinal Chemistry Department, Theodor Bilharz Research Institute, Kornaish El Nile, Warrak El-Hadar, Imbaba (P.O. 30), Giza 12411, Egypt; 2 Institute of Pharmacy and Molecular Biotechnology, Heidelberg University, 44883-2462 Heidelberg, Germany; sobeh@uni-heidelberg.de; 3 AgroBioSciences Research Division, Mohammed VI Polytechnic University, Lot 660–Hay MoulayRachid, 43150 Ben-Guerir, Morocco; 4 Pharmacology Department, Theodor Bilharz Research Institute, Kornaish El Nile, Warrak El-Hadar, Imbaba (P.O. 30), Giza 12411, Egypt; w.elmadawy@tbri.gov.eg (W.H.E.-M.); s.botros@tbri.gov.eg (S.B.); 5 Department of Pharmacognosy, Faculty of Pharmacy (Girls), Al-Azhar University, Cairo 11311, Egypt; halash1977@hotmail.com; 6 Pathology Department, Theodor Bilharz Research Institute, Kornaish El Nile, Warrak El-Hadar, Imbaba (P.O. 30), Giza 12411, Egypt; Seif200731@gmail.com

In the original publication [1], there was a mistake in Figure 5C “Kidney tissue of CP-treated mice”, as published. The authors apologize for any inconvenience caused by this oversight. These errors do not affect the results and conclusions published in the article. The new Figure 5 appears below. The authors state that the scientific conclusions are unaffected.

## Figures and Tables

**Figure 5:**
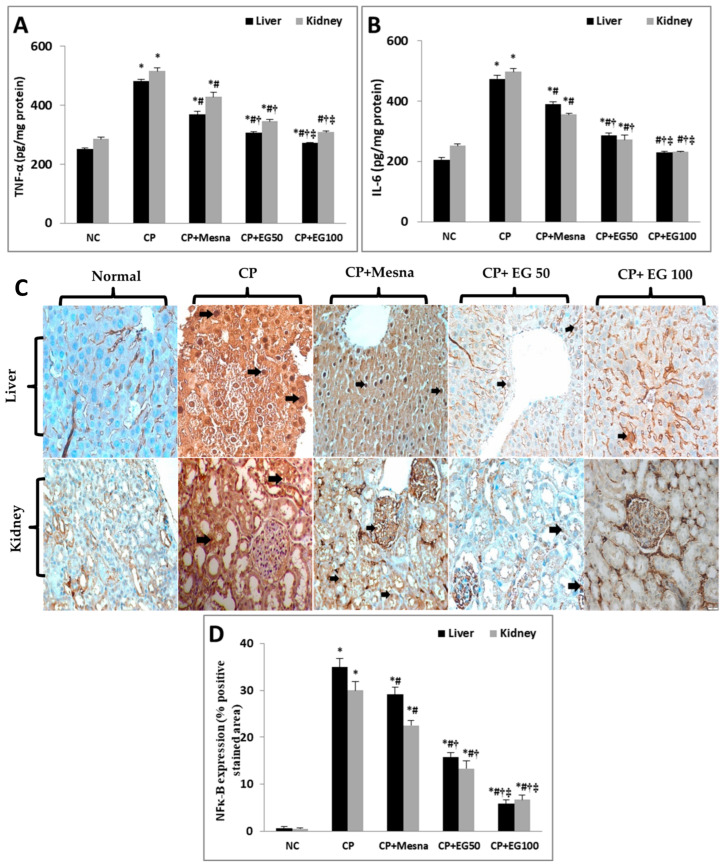
Effect of EG pretreatment on liver and kidney levels of pro-inflammatory markers (TNF-α (**A**), IL-6 (**B**)), IHC expression of NF-κB (×400, black arrows represent localization of positively stained brown nuclei with faint ignored background staining) (**C**) and semi-quantitative estimation of NF-κB positively stained nuclei in hepatic and renal tissues of CP-treated mice.
