# Supplementary material for: Chemical Profiling of Polyphenolics in Eucalyptus globulus and Evaluation of Its Hepato–Renal Protective Potential Against Cyclophosphamide Induced Toxicity in Mice
Source: Antioxidants (Basel). 2019 Sep 19;8(9):415. doi: 10.3390/antiox8090415 (PMC6769961; doi:10.3390/antiox8090415)
Supplement: Supplementary file 1 [file antioxidants-08-00415-s001.pdf]

antioxidants

Supplementary Material

Chemical Profiling of Polyphenolics in *Eucalyptus globulus* and Evaluation of Its Hepato-Renal Protective Potential Against Cyclophosphamide Induced Toxicity in Mice

Mosad A. Ghareeb [1,*], Mansour Sobeh [2,6], Walaa H. El-Maadawy [3], Hala Sh. Mohammed [4], Heba Khalil [5], Sanaa Botros [3] and Michael Wink [2,*]

[1] Medicinal Chemistry Department, Theodor Bilharz Research Institute, Kornaish El Nile, Warrak El-Hadar, Imbaba (P.O. 30), Giza 12411, Egypt
[2] Institute of Pharmacy and Molecular Biotechnology, Heidelberg University, 44883-2462 Heidelberg, Germany
[3] Pharmacology Department, Theodor Bilharz Research Institute, Kornaish El Nile, Warrak El-Hadar, Imbaba (P.O. 30), Giza 12411, Egypt
[4] Department of Pharmacognosy, Faculty of Pharmacy (Girls), Al-Azhar University, Cairo **11311**, Egypt
[5] Pathology Department, Theodor Bilharz Research Institute, Kornaish El Nile, Warrak El-Hadar, Imbaba (P.O. 30), Giza 12411, Egypt_
[6]AgroBioSciences Research Division, Mohammed VI Polytechnic University, Lot 660–Hay MoulayRachid, 43150 Ben-Guerir, Morocco
* Correspondence: m.ghareeb@tbri.gov.eg; +20201012346834 (M.A.G.); wink@uni-heidelberg.de; +49 (0)6221 54-4880 (M.W.)

Table of Contents	Page
**S1:** Annotation of the polyphenolic compounds using HPLC-DAD-ESI-MS/MS	2
**S2:** Structural elucidation of the isolated phenolic compounds	4

Annotation of The Polyphenolic Compounds Using HPLC-DAD-ESI-MS/MS (S1)

Phenolic Acids and Their Derivatives

Compound 3 was detected at *Rt* 1.41 min, it showed a deprotonated ion $[M-H]^-$ at *m/z* 169 and a diagnostic fragment ion at *m/z* 125 corresponding to the loss of CO_2 moiety (-*m/z* 44 u) $[M-H-CO_2$ moiety$]^-$, it could be identified as gallic acid [34]. **Compound 4** was detected at *Rt* 6.69, it showed a deprotonated ion $[M-H]^-$ at *m/z* 183 and MS^n ions were observed at *m/z* 169, 168, 125, 109, and 93, it was tentatively identified as methylgallate [35].

Flavonoids

Compound 12 was detected at *Rt* 18.68 min, it showed a deprotonated molecule $[M-H]^-$ at *m/z* 491 and MS fragment ion at *m/z* 315 [M-H-glucuronide moiety]$^-$ corresponding to the loss of glucuronide moiety (-*m/z* 176 u) was assigned to isorhamnetin aglycone, and key fragment ions for aglycone were observed at *m/z* 271, 179 and 151. Thus, compound 12 could be identified as isorhamnetin 3-*O*-β-D-glucuronoside [36]. **Compound 18** was detected at *Rt* 25.31 min, it showed a deprotonated molecule $[M-H]^-$ at *m/z* 329, and MS^n ions at *m/z* 314 $[M-H-CH_3]^-$, 299 $[M-H-2CH_3]^-$, 285 $[M-H-CO_2]^-$ and 243 $[M-H-C_2H_2O-CO_2]^-$, thus it could be identified as quercetin-3,4'-dimethyl ether [37,38]. Compound 22 was detected at *Rt* 40.34 min, it showed a deprotonated molecule $[M-H]^-$

at *m/z* 303, and MS^n ions were obtained at *m/z* 301, 285, 179, 177, and 125, it could be identified as dihydroquercetin (taxifolin) [39].

Hydrolyzable Tannins (Gallotannins and Ellagitannins)

Compound 7 was detected at *Rt* 13.55 min, it showed a deprotonated molecule $[M-H]^-$ at *m/z* 537 and MS^n fragment ions at *m/z* 385 $[M-H-152]^-$ due to the loss of galloyl moiety (-152 u), 313 $[M-H-224]^-$, a diagnostic ion was observed at *m/z* 271 $[M-H-266]^-$, this fragmentation pattern was typically assigned to 6S,9R-ionone 9-*O*-(6'-*O*-galloyl)-β-D-glucopyranoside (Mallophenol B) [40]. **Compound 9** was detected at *Rt* 16.29 min, it showed a deprotonated molecule $[M-H]^-$ at *m/z* 483, producing daughter ion at *m/z* 271 $[M-H-212]^-$ due to the total loss of galloyl moiety (-*m/z* 152 u), water molecule (-*m/z* 18 u) and another fragment (-*m/z* 42 u), characteristic ions for galloylglucose derivatives were observed at *m/z* 331, 313, 169, 151 and 137, thus it could be assigned to digalloylglucose, this compound was previously identified in both of Algerian *Eucalyptus globulus* fruits [41] and Egyptian *Eucalyptus camaldulensis* leaves [42]. **Compound 10** was detected at *Rt* 17.16 min, it showed a deprotonated molecule $[M-H]^-$ at *m/z* 421, producing daughter ion at *m/z* 169 [M-H-benzyl moiety-glucosyl moiety (Σ-*m/z* 252 u)]⁻ due to the loss of benzyl and glucose moieties. Other MS^n fragment ions were observed at *m/z* 331 due to the neutral loss of benzyl moiety (-*m/z* 90 u) [M-H-benzyl moiety]⁻, also a fragment ion was observed at *m/z* 313 due to the loss of water molecule (-*m/z* 18 u) from [M-H-benzyl moiety-H_2O]⁻, it could be identified as benzyl-galloylglucose [42]. **Compound 11** was detected at *Rt* 17.50 min, it showed a deprotonated molecule $[M-H]^-$ at *m/z* 481 and characteristic MS^n fragment ions for digalloylglucose nucleus at *m/z* 463 [M-H-H_2O (-*m/z* 18 u)]⁻, 329 [M-H-galloyl moiety (-*m/z* 152 u)]⁻, 301 [M-H-H_2O-glucose moiety (-*m/z* 180 u)]⁻, 313 211, and 169; therefore, it could be assigned to hexahydroxydiphenoyl-glucose (HHDP-glucose) [42]. Compound 13 was detected at *Rt* 19.64 min, it showed a deprotonated molecule $[M-H]^-$ at *m/z* 689 and it's a diagnostic fragment ion at *m/z* 537 $[M-H-152]^-$, which corresponds to the loss of a galloyl moiety, it can be interpreted as galloyl cypellocarpin B [42]. **Compound 14** was detected at *Rt* 19.82 min, it showed a deprotonated molecule $[M-H]^-$ at *m/z* 939 and a daughter ion at *m/z* 769 corresponding to loss of gallic acid moiety $[M-H-170]^-$, in addition to the appearance of MS^n fragment ions at *m/z* 787 and 635 which corresponds to the loss of the first galloyl moiety $[M-H-152]^-$, and the second one $[M-H-304]^-$ respectively, accordingly this compound can be interpreted as pentagalloylglucose [41]. **Compound 15** was detected at *Rt* 20.12 min, it showed a deprotonated molecule $[M-H]^-$ at *m/z* 635 and a daughter ion as a base peak at *m/z* 483 corresponding to the loss of galloyl moiety $[M-H-152]^-$, also diagnostic MS^n fragment ions were observed at *m/z* 465 $[M-H-170]^-$ and 423 $[M-H-212]^-$ due to the loss of gallic acid moiety, and the release of another galloyl moiety followed by cross ring fragmentation of glucose, 331 $[M-H-152-152]^-$, 313 $[M-H-152-152-18]^-$, 271 $[M-H-212-152]^-$ due to the loss of galloyl moieties, and water molecule; it could be interpreted as trigalloylglucose [41]. **Compound 16** was detected at *Rt* 21.54 min, it showed a deprotonated molecule $[M-H]^-$ at *m/z* 625 and a daughter ion at *m/z* 473 corresponding to the loss of galloyl moiety $[M-H-152]^-$, furthermore diagnostic MS^n fragment ions were appeared at *m/z* 607 $[M-H-H_2O]^-$ due to the loss of water molecule (-*m/z* 18 u), 437 $[M-H-18-170]^-$ corresponding to the loss of water and gallic acid moieties, thus compound could be characterized as HHDP-diglucoside [43]. **Compound 17** was detected at *Rt* 24.91 min, it showed a deprotonated molecule $[M-H]^-$ at *m/z* 629 and MS^n fragment ions at *m/z* 477 [M-H-galloyl moiety]⁻ due to the loss of galloyl moiety (-*m/z* 152 u) and corresponding to methylellagic acid glucoside, *m/z* 315 [M-H-galloyl glucoside moiety]⁻ due to the loss of galloyl glucoside moiety (-*m/z* 314 u) and corresponding to methyl ellagic acid, and a base peak at *m/z* 301 which corresponding to ellagic acid nucleus, this fragmentation pattern was typically assigned to galloyl ester of a methylellagic acid glucoside [41]. **Compound 19** was detected at *Rt* 27.17 min, it showed a deprotonated molecule $[M-H]^-$ at *m/z* 1085 and a diagnostic fragment ions at *m/z* 765 $[M-H-320]^-$, 633 $[M-H-452]^-$ due to the loss of trigalloyl moieties (-*m/z* 452 u) which corresponding to HHDP moiety, so it could be identified as eucalbanin A or its isomer cornusiin B [41]. **Compound 20** was detected at *Rt* 28.52 min, it showed a deprotonated molecule $[M-H]^-$ at *m/z* 519 and fragment ions at *m/z* 353 $[M-H-166]^-$, 335 $[M-H-184]^-$ due to the loss of oleuropeic acid

moiety (-*m/z* 184 u), and 233 [M-H-286]$^-$, this compound could be identified as cypellocarpin C [41]. **Compound 21** was detected at *Rt* 29.61 min, it showed a deprotonated molecule [M-H]$^-$ at *m/z* 1415 and fragment ions at *m/z* 1113 [M-H-302]$^-$ due to the loss of one HHDP moiety (-*m/z* 302 u), 933 [M-H-482]$^-$, 783 [M-H-632]$^-$ and 633 [M-H-782]$^-$, this compound could be characterized as Di (HHDP-galloylglucose)-pentose [41]. **Compound 23** was detected at *Rt* 41.82 min, it showed a deprotonated molecule [M-H]$^-$ at *m/z* 617 and fragment ions were appeared at *m/z* 465 [M-H-152]$^-$, 393 [M-H-224]$^-$, 317 [M-H-300]$^-$, 241 [M-H-376]$^-$ and 169 [M-H-448]$^-$, these fragmentation pattern was assigned to trigalloyllevoglucosan [44]. **Compound 24** was detected at *Rt* 53.13 min, it showed a deprotonated molecule [M-H]$^-$ at *m/z* 953 and MS^n fragment ions at *m/z* 635, 301 and 169, this fragmentation pattern was assigned to valoneoyl-digalloyl-glucopyranose. **Compound 26** was detected at *Rt* 56.04 min, it showed a deprotonated molecule [M-H]$^-$ at *m/z* 469 as well as MS^n fragment ions at *m/z* 425 [M-H-CO_2 (-*m/z* 44 u)]$^-$, 301 [M-H-168]$^-$ and 169 [M-H-300]$^-$, it was tentatively identified as valoneic acid dilactone [41].

Structural Elucidation of The Isolated Phenolic Compounds (S2)

Compound 1 was isolated as off-white amorphous powder, observed as faint violet fluorescence under short UV light, which turned to deep blue colour with $FeCl_3$ spray reagent. Molisch test was positive indicating a gallic acid glycoside. Acid hydrolysis afforded gallic acid in organic layer and rhamnose in aqueous layer. ^{1}H-NMR spectral data (400 MHz, DMSO-d_6) δ_H ppm: two symmetrical aromatic protons were resonated at δ_H 6.99 ppm (2H, s, H-2 and H-6), anomeric proton of sugar moiety at δ_H 4.91 ppm (1H, d, *J*=7.44 Hz) and remaining sugar protons at δ_H 3.17-3.74 ppm (m). ^{13}C-NMR spectral data (100 MHz, DMSO-d_6), carbon atoms of gallic acid nucleus were resonated at δ_C 167.9 (CO), 145.8 (C-3, C-5), 138.4 (C-4), 120.9 (C-1), 109.1 ppm (C-2, C-6). While, carbon atoms of *O*-β-xylopyranosyl moiety were resonated at δ_C 103.3 (C-1'), 76.8 (C-3'), 73.6 (C-2'), 69.8 (C-4') & 65.6 ppm (C-5'). Based on the above mentioned chromatographic properties, NMR data and literature [45], compound 3 was identified as 4-(*O*-β-D-xylopyranosyloxy)-3,5-di-hydroxy-benzoic acid (Gallic acid pentoside).

Compound 2 was isolated as off-white amorphous powder, observed as faint violet fluorescence under short UV light, which turned to deep blue colour with $FeCl_3$ spray reagent. Molisch test was positive indicating a gallic acid glycoside. Acid hydrolysis afforded gallic acid in organic layer and rhamnose in aqueous layer. ^{1}H-NMR spectral data (400 MHz, DMSO-d_6), two symmetrical aromatic protons were resonated at δ_H 6.97 ppm (2H, s, H-2 and H-6), anomeric proton of sugar moiety at δ_H 4.63 ppm (1H, brs, H-1'), remaining sugar protons were resonated at δ_H 3.25-3.55 ppm (m) and aliphatic methyl protons at δ_H 1.09 ppm (3H, d, *J*= 6.8 Hz, CH_3-C-6'). ^{13}C-NMR spectral data (100 MHz, DMSO-d_6), carbon atoms of gallic acid nucleus were resonated at δ_C 167.9 (COO), 145.8 (C-3, C-5), 138.4 (C-4), 120.8 (C-1), and 109.1 ppm (C-2 & C-6). On the other hand, carbon atoms of *O*-α-L- rhamnopyranosyl moiety were resonated at δ_C 104 (C-1'), 72.7 (C-4'), 70.3 (C-3 & 4), 69.4 (C-5'), and 18.9 ppm (CH_3-6'). Based on the above mentioned chromatographic properties, NMR data and literature [45], compound 4 was identified as 4-(*O*-α-L-rhamnopyranosyloxy)-3,5-di-hydroxy-benzoic acid (Gallic acid rhamnoside).

Compound 3 was isolated as off-white amorphous powder, observed as faint violet fluorescence under short UV light, which turned to deep blue colour with $FeCl_3$ spray reagent. UV spectral data showed a characteristic band at λ_{max} = 272 nm. ^{1}H-NMR spectral data (400 MHz, DMSO-d_6), revealed the presence of two symmetrical protons located in the aromatic region at δ_H 6.94 ppm (2H, s, H-2 and H-6). ^{13}C-NMR spectral data (100 MHz, DMSO-d_6) showed a set of aromatic carbons were resonated at δ_C 109.1 ppm (C-2, C-6), 120.8 ppm (C-1), 138.4 ppm (C-4), 145.8 ppm (C-3, C-5) and carbonyl carbon at δ_C 167.9 ppm (-CO) [46]. Based on the above mentioned NMR data, comparison with authentic samples (CO-PC) and literature, compound 1 was identified as 3,4,5-trihydroxy-benzoic acid (gallic acid).

Compound 4 was isolated as off-white amorphous powder, observed as faint violet fluorescence under short UV light, which turned to deep blue colour with $FeCl_3$ spray reagent. UV spectral data showed a characteristic band at λ_{max} = 272 nm. ^{1}H-NMR spectral data (400 MHz,

DMSO-d_6), showed two symmetrical protons in the aromatic region at δ_H 6.91 ppm (2H, brs, H-2 and H-6), another characteristic signal for aliphatic methoxy protons was observed at δ_H 3.7 ppm (3H, s, -OCH_3).^{13}C-NMR spectral data (100 MHz, DMSO-d_6), showed three types of carbon signals including oxygenated aliphatic carbon at δ_C 51.68 ppm (OCH_3), a set of aromatic carbon signals were observed at δ_C 108.57 ppm (C-2, C-6), 119.36 ppm (C-1), 138.48 ppm (C-4), 145.56 ppm (C-3, C-5) and carbonyl carbon was detected at δ_C 166.41 ppm (-CO) [47,48]. Based on the above mentioned chromatographic properties, NMR data, comparison with authentic samples (CO-PC) and literature, compound 2 was identified as methyl 3,4,5-trihydroxybenzoate (methyl gallate).
